# Sub-communities of the vaginal microbiota in pregnant and non-pregnant women

**DOI:** 10.1098/rspb.2023.1461

**Published:** 2023-11-29

**Authors:** Laura Symul, Pratheepa Jeganathan, Elizabeth K. Costello, Michael France, Seth M. Bloom, Douglas S. Kwon, Jacques Ravel, David A. Relman, Susan Holmes

**Affiliations:** ^1^ Department of Statistics, Stanford University, 390 Jane Stanford Way, Stanford, CA 94305, USA; ^2^ Department of Mathematics and Statistics, McMaster University, 1280 Main Street, West Hamilton, Ontario, Canada L8S 4K1; ^3^ Department of Medicine, Stanford University School of Medicine, 300 Pasteur Drive, Stanford, CA 94305, USA; ^4^ Institute for Genome Sciences, University of Maryland School of Medicine, 670 W. Baltimore Street, Baltimore, MD 21201, USA; ^5^ Department of Microbiology and Immunology, University of Maryland School of Medicine, 685 West Baltimore Street, HSF-I Suite 380, Baltimore, MD 21201, USA; ^6^ Division of Infectious Diseases, Massachusetts General Hospital, 55 Fruit Street, Boston, MA 02114, USA; ^7^ Harvard Medical School, 25 Shattuck St, Boston, MA 02115, USA; ^8^ Ragon Institute of MGH, MIT, and Harvard, 400 Technology Square, Cambridge, MA 02139, USA; ^9^ Department of Microbiology & Immunology, Stanford University School of Medicine, 299 Campus Drive, Stanford, CA 94305, USA; ^10^ Infectious Diseases Section, Veterans Affairs Palo Alto Health Care System, 3801 Miranda Avenue, Palo Alto, CA 94304, USA

**Keywords:** vaginal microbiota, multi-omics, menstrual cycle, pregnancy

## Abstract

Diverse and non-*Lactobacillus*-dominated vaginal microbial communities are associated with adverse health outcomes such as preterm birth and the acquisition of sexually transmitted infections. Despite the importance of recognizing and understanding the key risk-associated features of these communities, their heterogeneous structure and properties remain ill-defined. Clustering approaches are commonly used to characterize vaginal communities, but they lack sensitivity and robustness in resolving substructures and revealing transitions between potential sub-communities. Here, we address this need with an approach based on mixed membership topic models. Using longitudinal data from cohorts of pregnant and non-pregnant study participants, we show that topic models more accurately describe sample composition, longitudinal changes, and better predict the loss of *Lactobacillus* dominance. We identify several non-*Lactobacillus*-dominated sub-communities common to both cohorts and independent of reproductive status. In non-pregnant individuals, we find that the menstrual cycle modulates transitions between and within sub-communities, as well as the concentrations of half of the cytokines and 18% of metabolites. Overall, our analyses based on mixed membership models reveal substructures of vaginal ecosystems which may have important clinical and biological associations.

## 1. Introduction

Several critical aspects of women's health are linked to the structure of the vaginal microbiota [1–3]. Vaginal microbiotas dominated by beneficial *Lactobacillus* species are associated with positive health outcomes [3]. A paucity of *Lactobacillus* and a diverse array of strict and facultative anaerobes, however, are associated with negative health outcomes such as preterm birth [4,5] and susceptibility to sexually transmitted infections [6–9], including HIV [10–12]. Longitudinal studies of vaginal microbiota composition have revealed its dynamic nature [4,13,14]. In non-pregnant individuals, a virtually complete replacement of the microbiota is sometimes observed, typically around the time of menses [13,15]. While complete replacement is rare, more modest (i.e. of a fraction of the microbiota composition), or slower (i.e. over a few days or weeks) changes in composition are relatively common in both pregnant and non-pregnant individuals [4,13,14]. The microbiota of pregnant women may appear more stable than that of non-pregnant individuals; however, differences in sampling frequencies might not allow us to fully characterize the differences in microbiota dynamics. Non-*Lactobacillus*-dominated microbiotas are generally less stable than *Lactobacillus*-dominated ones [4,13,14]. Some *Lactobacillus* species, such as *L. crispatus*, better resist replacement by non-*Lactobacillus* species and create greater vaginal ecosystem stability during and outside pregnancy [13,14,16]. By contrast, *L. iners* is more frequently associated with non-optimal communities [13,14,16]. Non-optimal vaginal microbiotas (i.e. non-*Lactobacillus*-dominated microbiotas) are typically highly heterogeneous within and between individuals [4,13,14]. It remains, however, poorly understood whether non-optimal microbiota composition is random (i.e. individual-specific) or composed of distinct sub-communities (i.e. consortia of bacteria interacting with each other). If such sub-communities do exist, it remains to be seen whether they are differentially associated with characteristics of the host or with specific negative health outcomes.

**Figure 1.**
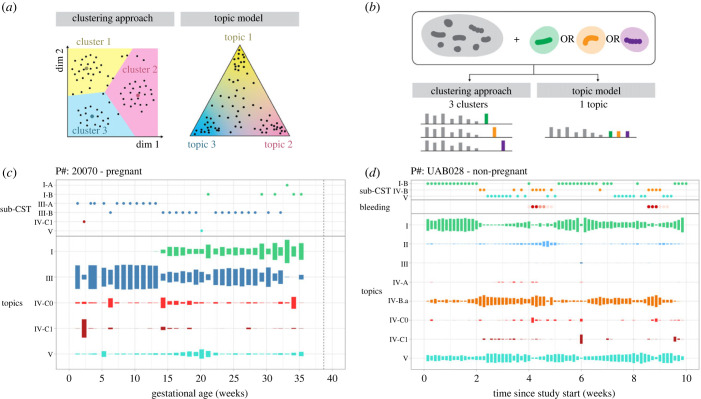
Topic models are mixed membership models and reveal transitions between states. (*a*) Schematics contrasting sample characterization in a lower dimensional space by clustering methods versus topic models. In both schematics, each dot is a sample. Larger coloured dots in the clustering schematic indicate centroids. (*b*) Schematic illustrating how clustering versus topic models would capture a ‘functional equivalence’ phenomenon. Two or more species are potentially ‘functionally equivalent’ if they occupy the same ecological niche (thrive in similar environments and with other species) but rarely co-occur because they may compete for the same resources. (*c–d*) Examples of time-series displays of changes in microbiota composition summarized by cluster membership (sub-CST—top) or topic proportions (bottom) in a (*c*) pregnant and (*d*) non-pregnant participant. Topics were labelled such that their name matched the (sub)CST with the most similar composition (figure 2*c*). The height of the topic rectangles codes for the proportion of that topic in samples. Their proportion for a given sample sums to 1.

Efforts to address these questions have so far relied on clustering approaches. Various clustering methods are commonly applied to taxonomic abundance tables to define community structure. This has led to the adoption of the concepts of community state types (CSTs) or community types (CTs) [17,18]. More recently, in order to define ‘reference sub-CSTs’ (i.e. dataset- or study-independent), large composite datasets have been clustered, and several non-*Lactobacillus*-dominated clusters (sub-CSTs) have been identified across populations of non-pregnant women [19]. While clustering serves as a useful dimensionality reduction tool for describing complex microbiota compositions, it may fail to capture clinically relevant structures. For example, two samples could belong to the same cluster (III-B) because they both show a bare majority of *L. iners* (e.g. 60%), but be accompanied by *L. crispatus* in one case, and by a diverse panel of non-*Lactobacillus* species in the other case, which may have different health implications. In addition, clustering-based approaches fail to model *transition* or *intermediary states* between clusters (figure 1). Modelling *transitions* is especially important in the context of the vaginal microbiota as its composition may change several times over a few months, weeks, or even a few days, as observed in menstruating individuals [4,13–15]. However, because samples are assigned to a single cluster (figure 1*a*), transitions between clusters may appear identical (i.e. described by the same sequence of clusters) while the underlying microbiota trajectories were drastically different in rate (progressive versus abrupt) or in the nature of the intermediate compositions. Finally, while clustering approaches can identify sets of species that frequently co-occur, they are not well suited to identify subsets of species that may have similar functions but not frequently found together (figure 1*b*). The discrepancies between the clustering assumptions and our understanding of the composition and dynamics of the vaginal microbiota highlight the need for better-suited dimension reduction statistical models.

Topic models, first developed to infer population structure [20] and later formally described as latent Dirichlet allocation (LDA) in the context of natural language processing [21], have recently been proposed for analysing microbial communities and identifying sub-communities [22]. Unlike clustering-based categorization, where samples are assigned to a single category, samples are modelled as mixtures of topics (sub-communities), and each topic is characterized by a particular distribution of bacterial species. For example, if a sample were described as 70% topic 1 and 30% topic 2, this would mean that the species subsumed in topic 1 accounted for 70% of the sample, while the species in topic 2 accounted for the remaining 30%. Topics may be sparse or include a larger number of species and some species may belong to several topics. In addition to more realistically model microbiota composition, topic models do not require any normalization of the count tables (typically the number of 16S rRNA genes sequenced in each sample) as they are hierarchical Bayesian models that explicitly account for library sizes.

Here, we sought to deepen our understanding of the fine structure of non-optimal vaginal microbiotas by applying topic models to longitudinal samples acquired from pregnant and non-pregnant women. We compared them to previously identified reference clusters and investigated the clinical relevance of the identified sub-communities and their association with host characteristics, pregnancy status, the risk of preterm birth, or the phase of the menstrual cycle. The menstrual cycle effects on the vaginal ecosystem were further evaluated by identifying vaginal metabolites (both host- and bacteria-produced) and cytokines (host-produced) with differential abundances throughout the cycle.

## 2. Results

### (a) Topic analysis identifies nine sub-communities in the vaginal microbiota of pregnant and non-pregnant women

We analysed data from 2179 vaginal samples collected weekly from 135 pregnant individuals enrolled at two sites in the USA (Stanford University, Stanford, CA, USA and University of Alabama, Birmingham, AL, USA) and 1534 vaginal samples collected daily from 30 non-pregnant individuals enrolled at the University of Alabama, Birmingham (see Material and methods; see electronic supplementary material, table S1 for demographic data). Topic models were fit to the count data of 16S rRNA amplicon sequence variants (ASVs) agglomerated by taxonomic assignment.

**Figure 2.**
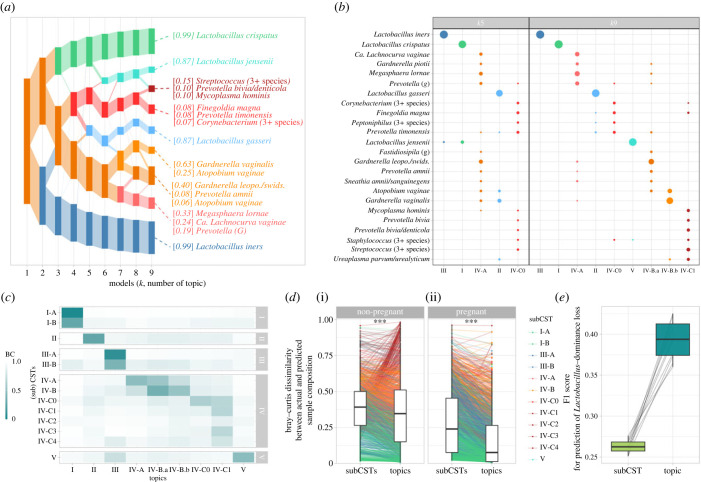
Sub-communities identified by topic models. (*a*) Alignment of topics (rectangles) for models fitted with an increasing number of topics (*x*-axis). The rectangle's height scales with the total proportion of the corresponding topic in all samples: taller rectangles represent more prevalent topics. Topics are connected across models (*x*-axis) according to their alignment weights, which reflect their similarities (see Material and methods). Topics of the *k* = 9 model are annotated with their most prevalent species, and the numbers in brackets indicate the proportion of that species in the topic. Annotations included the three most prevalent species that made up at least 5% of the topic composition. (*b*) Topic composition for *k* = 5 (coarse representation, left) or *k* = 9 (optimal tradeoff between dimension reduction and descriptive accuracy, right) topics. The proportion of each species (*y*-axis) within each topic (*x*-axis) is encoded by the dot size. Proportions sum to 1 for each topic. For readability and conciseness, species were included if they accounted for at least 0.5% of a topic. (*c*) Comparison of the topics (*x*-axis) and sub-CSTs (*y*-axis) compositions. Topics and sub-CSTs with similar compositions are characterized by a low Bray–Curtis dissimilarity and a darker hue. (*d*) Bray–Curtis dissimilarity between actual and predicted sample composition (*y*-axis) by sub-CSTs or topics (*x*-axis) in non-pregnant (i) and pregnant (ii) individuals. Each line is a sample, coloured by its sub-CST membership. Stars indicate statistical significance of a one-sided paired *t*-test (***p < 0.001). (*e*) F1 scores (harmonic mean of precision and sensitivity, *y*-axis) for the prediction of Lactobacillus dominance loss (i.e. total proportion of Lactobacillus falling below 50%) at the next sample when predicted from sub-CST membership (light green) or topic memberships (dark turquoise). Distributions were obtained from 10 independent training-testing sets (electronic supplementary material, methods). Thin lines connect F1 scores from the same training-testing set.

Topic analysis requires choosing K, the number of topics, which can be estimated using cross-validation or, as recently proposed [23], by performing topic alignment across models with different resolutions (i.e. with different K; figure 2*a*). In contrast to cross-validation, this latter approach shows how topics at higher resolution relate to topics at lower resolution and provides several diagnostic scores. These scores characterize each topic across degrees of resolution and allow us to evaluate deviations from the LDA assumptions. Here, topic alignment suggested that nine topics provided the best compromise between dimension reduction and accurate modelling of taxonomic counts (electronic supplementary material, methods; figure 2*a,b*). If a coarser resolution were desired, the alignment refinement scores suggested that *K* = 5 topics would be the most suited as topics at higher resolutions were sub-topics of these five topics (electronic supplementary material; figure 2*b*).

At *K* = 9, four of these nine topics were dominated by one of the four most common *Lactobacillus* spp. (*L. crispatus*, *L. gasseri*, *L. iners* and *L. jensenii*; figure 2*a,b*). The composition of the five remaining topics did not include any *Lactobacillus* spp. (figure 2*a,b*). These five non-Lactobacillus topics could be grouped into two groups based on the alignment: one group contained three topics which included *Gardnerella*, *Atopobium* and *Megasphaera* spp., while the other group contained *Finegoldia*, *Corynebacterium* and *Streptococcus* (figure 2*a,b*).

### (b) Topics provide a more succinct, yet more accurate, description of microbiota composition than sub-CSTs

To evaluate the generalizability of the identified sub-communities, we compared the topic composition with the composition of the 12 ‘reference’ sub-CSTs (Valencia centroids) previously identified in a composite dataset of non-pregnant individuals' samples [19] (figure 2*c*). To compare topics and clusters, we computed the Bray–Curtis dissimilarities between their compositions after harmonizing taxonomic assignments (figure 2*c*; electronic supplementary material, methods). Topics were labelled to match their most similar (sub-)CST (Material and methods; figures 1*c,d* and 2*b*). The comparison showed that two *L. crispatus*-dominated sub-CSTs (I-A and I-B) have high similarity with the single *L. crispatus*-dominated topic (I). Similarly, two *L. iners*-dominated sub-CSTs (III-A and III-B) match a single *L. iners*-dominated topic (III). This is because CST I-A and I-B (or III-A and III-B) describe microbiotas that are either fully dominated by *L. crispatus* (subCST I-A) or *L. iners* (subCST III-A) versus those partially dominated by *L. crispatus* or *L. iners* and hosting other species (sub-CST I-B or III-B). By contrast, because topic models allow samples to be composed of several topics, a single topic is sufficient to account for *L. crispatus* (topic I) or *L. iners* (topic III) counts. Samples in which *L. crispatus* co-exists with *L. iners* will be represented by a mix of topics I and III, while a sample where *L. crispatus* co-exists with a *Gardnerella* species by a mix of topics I and IV-A/B. CST II and V have a one-to-one optimal match with topics II and V.

When comparing non-*Lactobacillus* sub-CSTs and topics, we observed that (i) sub-CST IV-A and IV-B are represented by three topics (IV-A, IV-B.a and IV-B.b), which can, in part, be explained by differences in taxonomic assignment used for topics (e.g. *Gardnerella* species are undifferentiated in sub-CSTs, while, here, some *Gardnerella* ASVs were matched to different species—see electronic supplementary material, methods), and (ii) a single topic (IV-C1) matches four sub-CSTs (IV-C1 – IV-C4). This is because these four sub-CSTs only differ in the proportion of four seemingly mutually exclusive genera (*Streptococcus*, *Enterococcus*, *Bifidobacterium* and *Staphylococcus*), with one of these four genera dominating each sub-CST; the prevalence of the remaining genera or species is similar across the four IV-C1-4 sub-CSTs (electronic supplementary material, figure S1). In our data, we also observed rare co-occurrence of these four genera (electronic supplementary material, figure S2–S3). In the presence of such mutual exclusion, clustering approaches tend to create several clusters; by contrast, because topic models allow for synonyms, topic IV-C1 embeds these species within a single topic, as illustrated in figure 1*b*.

We next examined three potential benefits of using topic mixed memberships instead of clustering categorization (sub-CSTs). Our first conjecture was that topics would provide a more accurate representation of sample compositions than sub-CSTs. The second was that this effect would be primarily driven by samples from unstable microbiotas. Our third conjecture held that topic memberships would better predict whether an individual is at risk of losing *Lactobacillus* dominance at the next time-point.

To test our first conjecture (i.e. accuracy of representation), we compared the Bray–Curtis dissimilarity between the actual sample compositions and the sample compositions predicted by topic mixed memberships or by sub-CST membership. The predicted composition of a sample is either the composition of the centroid of the sample's sub-CST or the average topic composition (displayed in figure 2*b*) weighted by the proportion of each topic in that sample (Material and methods). The Bray–Curtis dissimilarity between actual and predicted sample composition was smaller when sample compositions were predicted by topics (figure 2*d*). This effect was stronger in pregnant participants (mean difference = 0.12, paired *t*-test *p*-value < 0.001) than in non-pregnant participants (mean difference = 0.02, *p*-value < 0.001). The smaller mean difference in non-pregnant women compared to pregnant women can partially be explained by samples belonging to sub-CSTs IV-C1-4. These samples were dominated by one of the four seemingly mutually exclusive species mentioned above (*Streptococcus*, *Enterococcus*, *Bifidobacterium* and *Staphylococcus*), considered synonyms in topic models, and found in a single topic. When omitting these samples, the mean difference in dissimilarity in non-pregnant women increased from 0.02 to 0.07 (electronic supplementary material).

Our second conjecture was that the composition of samples from stable microbiotas (i.e. their composition remains largely unchanged over time) would be equally well described by sub-CSTs or by topics because these microbiotas would have stabilized over robust sub-communities well captured by clustering approaches. By contrast, we expected that samples from unstable microbiotas would be better described by topic mixed memberships because the transitions between well-defined sub-communities can be better captured by varying memberships. Our results supported this expectation in pregnant participants, but not in non-pregnant participants (electronic supplementary material, figure S4). This was tested using the Bray–Curtis dissimilarities computed above and comparing their differences (sub-CSTs versus topics) in samples from stable versus unstable microbiotas. Samples were considered stable if they belonged to a group of at least five consecutive samples whose Bray–Curtis dissimilarity was less than 0.25 (similar results were obtained for 0.15 and 0.35—see electronic supplementary material, table S2) and were considered unstable otherwise. In pregnant participants, the mean difference in dissimilarities was 0.08 for samples from stable microbiotas and 0.14 for samples from unstable microbiotas (one-sided *t*-test *p*-value < 0.001). In non-pregnant participants, these differences were small and approximately the same in samples from both stable (0.03) and unstable (0.02) microbiotas.

We next evaluated our third conjecture: topic memberships would better identify individuals at risk of losing *Lactobacillus* dominance, defined here as overall *Lactobacillus* proportions falling below 50%. Past studies have shown that individuals whose microbiota is categorized as CST III (*L. iners*-dominated) are more at risk of losing *Lactobacillus* dominance than those in other *Lactobacillus*-dominated CSTs (I, II and V) [14,16] but this risk has not been evaluated with a more refined definition of microbiota composition. To do so, we trained and 10×-cross-validated logistic regression models to predict the loss of *Lactobacillus* dominance (Material and methods). Since only 11% of *Lactobacillus*-dominated microbiotas switch to non-*Lactobacillus*-dominated ones (i.e. we are predicting rare events), F1 scores (harmonic mean of precision and sensitivity) were used to compare prediction performances (figure 2*e*). Topic memberships better predicted the risk of losing *Lactobacillus* dominance than sub-CST (median F1 score of 0.4 versus 0.27, Wilcoxon test *p-*value < 0.002). Specifically, topic-based predictions are more precise (i.e. lower false positive rate) than sub-CST-based predictions (precision of 0.26 versus 0.16, *p-*value < 0.002, electronic supplementary material, figure S5).

Given these results and the three advantages conferred by topic models, we next explored the demographic associations and functional relevance of the identified sub-communities.

### (c) Topic composition varies with demographic characteristics and pregnancy status

**Figure 3.**
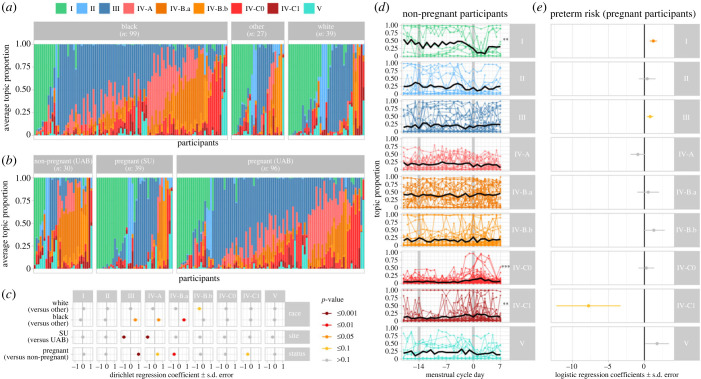
Sub-communities and demographic and reproductive characteristics. (*a–b*) Topic composition per racial group (*a*) or cohort (*b*). Vertical bars show the average proportion of each topic (colour) for each participant (*x*-axis), ordered by their most prevalent topic. (*c*) Dirichlet regression estimated coefficients (*x*-axis) quantifying the associations between race, study site, pregnancy status (*y*-axis) and topic proportions (horizontal panels). Colours indicate the statistical significance. (*d*) Topic proportions throughout the menstrual cycle (day 0 indicates the first day of menses—figure 4*a*). Each dot is a sample. Lines connect samples from the same participant and cycle. Thick black lines show the average topic proportions across all participants. Stars on the right indicate the statistical significance of the associations between topic proportions and menstrual cycle (***p < 0.001, **p < 0.01). (*e*) Logistic regression estimated coefficients (*x*-axis) quantifying the association between average topic proportion and preterm birth in pregnant individuals. Colours are as in (*c*).

Samples were collected from three cohorts: non-pregnant women recruited at the University of Alabama Birmingham in 2009–2010, pregnant women recruited at the same institution in 2013–2015, and pregnant women recruited at Stanford University in 2013–2015. Recruitment sites and participants' race were associated with differential proportions of several topics. The microbiotas of Black participants and participants recruited at UAB were more likely to contain topics III (*L. iners*-dominated), IV-A and IV-B.a (both non-*Lactobacillus*-dominated) (figure 3*a–c*). Topics III and IV-A were also more prevalent in pregnant participants, while topics IV-B.b and IV-C1 were less prevalent in non-pregnant participants (figure 3*a–c*).

### (d) Topics IV-C0 and IV-C1 increase during menses; topic IV-C1 is also associated with preterm birth

The proportions of both topics IV-C0 and IV-C1 increased during menses (*p*-values < 0.001 and 0.01 resp.; figure 3*c*). By contrast, the proportion of topic I (*L. crispatus*-dominated) decreased during menses (*p*-value < 0.01). Consistent with previous findings [4], topic I (*L. crispatus*-dominated) was associated with term delivery, while topic IV-C1 had a strong association with preterm delivery, although not passing the significance threshold (*p* = 0.051).

### (e) The menstrual cycle shapes the vaginal microbial composition

**Figure 4.**
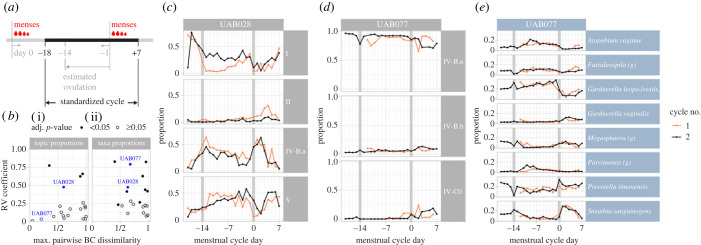
The menstrual cycle shapes microbial composition. (*a*) Schematic illustrating the features of standardized cycles. (*b*) Scatter plot, in which each dot is a participant, showing the RV coefficient of agreement (*y*-axis) between the proportions of topics (i) or taxa (ii) of a participant's consecutive cycles and (*x*-axis) the magnitude of change in microbiota composition throughout the cycle measured by the maximum of the pairwise Bray–Curtis dissimilarity between composition at each cycleday. Participants shown in *c*–*e* are highlighted in blue. (*c–d*) Topic composition of two participants with data available for at least two menstrual cycles (first in orange, second in black). The time-series display shows topic proportion (*y*-axis) on each cycle day (*x*-axis). Topics were included if their median proportion across cycles was higher than 1% and their maximal proportion higher than 5%. (*e*) The same display as in *d* but with the taxa proportions on the *y*-axis. Taxa with median proportion higher than 1% and maximal proportion higher than 10% were included.

Prompted by the observation that the proportions of several topics varied with the menstrual cycle, we investigated longitudinal associations between menstrual cycle and microbiota composition. Among the 30 non-pregnant participants, 26 had reported vaginal bleeding allowing the identification of at least one menstrual cycle and we had data over two consecutive cycles for 20 participants (Material and methods). Cycles were standardized from 18 days before menses to 7 days after first day of menses as the luteal phase (after ovulation) vary less in duration than the follicular phase (before ovulation) [24,25] (figure 4*a*; Material and methods).

When characterized by sub-CST membership, the vaginal microbiota structure of only 2/20 participants (10%) showed a statistically significant agreement between consecutive cycles (electronic supplementary material, figure S6) as measured by the RV coefficient (adj. *p*-value < 0.05, electronic supplementary material, methods). When characterized by topic mixed membership, that proportion doubled (20% - 4/20 participants; figure 4*b–d*). However, within-subcommunity changes were still frequent. Indeed, for six additional participants, although the topic proportions remained relatively stable throughout their cycle, the underlying taxa composition varied (e.g. participant UAB077; figure 4*d,e*). In total, half (10/20) of the participants had a statistically significant agreement between their taxa proportions in two consecutive cycles (figure 4*b*, right panel).

**Figure 5.**
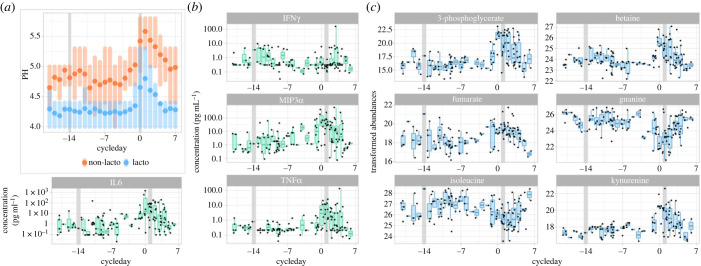
Vaginal pH, cytokines and metabolites throughout the menstrual cycle. (*a*) Distribution of vaginal pH throughout the menstrual cycle in Lactobacillus-dominated samples (blue) and non-Lactobacillus-dominated samples (orange). Dots indicate the means, shaded vertical bars span the 25th–75th percentiles. (*b,c*) Concentration (*y*-axis) of four cytokines (*b*) and six metabolites (*c*) with significant variations throughout the menstrual cycle (*x*-axis). Each dot is a sample.

Prompted by the observation that the menstrual cycle is associated with longitudinal variations of the microbiota composition, we further investigated whether the vaginal environment, characterized by pH values and vaginal metabolite and cytokine concentrations, also varied with the cycle. Consistent with past results [17], the vaginal pH of *Lactobacillus-*dominated samples (i.e. proportions of *Lactobacillus* >50%) was lower (4.4, 90% 4.0–5.3) than that of non-*Lactobacillus-*dominated samples (5.0, 90% 4.0–5.8). The pH remained stable throughout the cycle (*Lactobacillus*-dominated: 4.3, 90% 4.0–5.3; non-*Lactobacillus*-dominated: 4.9, 90% 4.0–5.5), except during menses when it increased by about 0.5 units in *Lactobacillus*-dominated (4.7, 90% 4.0–5.8) and non-*Lactobacillus*-dominated samples (5.4, 90% 4.4–7.0) (figure 5*a*).

Half of the cytokines (10 out of 20, *p*-values < 0.01, adjusted for multiple testing) showed a significant association with the menstrual cycle. Most cytokines (e.g. IL6 or TNF*α*) peaked during menses, while two of them (IFN*γ* and IL13) showed elevated abundance about the time of ovulation (figure 5*b*; electronic supplementary material, figure S7). In total, 18% of metabolites (60 out of 336) were also significantly associated with the menstrual cycle (figure 5*c*; electronic supplementary material, figure S8). Most (72%) had increased or decreased abundances in the late luteal phase or during menses (i.e. between cycle day −3 and 5; electronic supplementary material, figure S8).

## 3. Discussion

In this study, we used topic models, a mixed membership method, to identify bacterial sub-communities within vaginal microbiota samples from both pregnant and non-pregnant US women. We identified four *Lactobacillus*-dominated sub-communities corresponding to the four *Lactobacillus*-dominated CST, and five non-*Lactobacillus* sub-communities (i.e. topics), refining the structure of samples traditionally assigned to CST IV [17]. This CST (CST IV) is particularly relevant clinically as a paucity of *Lactobacillus* species is associated with bacterial vaginosis (BV), an increased risk of preterm birth, and a higher susceptibility to acquiring sexually transmissible infections [3–6,10–12,14].

These five non-*Lactobacillus* sub-communities were found to belong to two groups. One group contained three topics (IV-A, IV-B.a and IV-B.b) and characterized by the co-occurrence of species from the *Gardnerella*, *Megasphaera*, *Atopobium*, *Fastidiosipila* and *Sneathia* genera, and *Prevotella amnii*. The other group contained two topics (IV-C0 and IV-C1). This group contained species from the *Corynebacterium*, *Finegoldia*, *Peptoniphilus*, *Bifidobacterium*, *Staphylococcus* and *Streptococcus* genera, and *Prevotella bivia/denticola* and *timonensis*. These two groups of topics align with previously identified sub-groups resulting from clustering a large collated dataset of non-pregnant women samples [19]: sub-CST IV-A and B belong to the first group, and sub-CSTs IV-C0-4 to the second group. This study thus confirms that non-*Lactobacillus*-dominated microbiotas present sub-structures that may have clinical relevance.

The main difference between the approach used here (topic analysis) and clustering approaches traditionally used to identify sub-groups in the vaginal microbiota lies in the *mixed membership* nature of topic models, thereby allowing samples to be associated with multiple topics in different proportions. This property offers the advantage of revealing longitudinal transitions between sub-communities and the rate at which they occur, which is impossible with clustering approaches. We showed here that, in pregnant participants, stable microbiotas were almost equally well characterized by clusters and topics; by contrast, unstable microbiotas compositions were better represented by mixed topic memberships than by sub-CSTs. Topic memberships could also better predict the risk that a participant's microbiota would lose its *Lactobacillus* dominance and switch to a sub-optimal microbiota composition.

In this study, we compared topic- and clustering-based sample descriptions in cases in which sub-communities (mixed) memberships were used as explanatory variables; the actual microbiota composition or the risk of losing *Lactobacillus* dominance were our response variables. We expect that colleagues might also find advantages in using sub-community mixed memberships (topic-based sample description) as a *multivariate response variable* to identify host or intervention related factors associated with specific transitions or intermediate states. In contrast to univariate alternative or clustering, this might better reflect the potential multiple etiologies of vaginal dysbiosis.

Another difference between topic models and clustering approaches is that topic models allow for ‘synonyms’, which may reflect *potential functional equivalences* in a microbial community context. Indeed, if two species are found interchangeably (but not simultaneously) with a specific combination of other species, these two species will be found in the same topic. By contrast, clustering approaches tend to create two clusters (one containing each species) potentially artificially increasing the number of functionally relevant sub-communities. This matches our observations as a single topic encapsulates four sub-CSTs (IV-C1-4) [19] characterized by four mutually exclusive genera that co-occur with the same set of other species. In sub-community IV-C1 (and subCST IV-C1 – IV-C4), these four genera are *Streptococcus*, *Enterococcus*, *Bifidobacterium* and *Staphylococcus* and these sub-communities are found with higher prevalence in non-pregnant individuals, often during menses.

Topic models used in this study are unsupervised methods, and, like clustering, topic models identify dataset-specific features. This means that sub-communities identified in samples from a different cohort may differ from those identified in this study. However, we expect these sub-communities to be reproducibly observed in other (North American) populations since the sub-communities revealed by our analysis were found in individuals from three distinct cohorts, encompassing both pregnant and non-pregnant individuals from two distinct North American sites. Further, the agreement between our topics and the composition of ‘reference sub-CSTs’ previously identified in non-pregnant individuals [19] supports the generalizability of our findings. Deeper sequencing methods (e.g. metagenomics) may allow a more precise taxonomic characterization of microbiota samples and further refinement of these sub-communities.

To evaluate the functional or clinical relevance of these sub-communities, we performed a series of analyses to investigate the associations with demographic, clinical variables or outcomes. We found several significant associations between these subcommunities and the demographic characteristics or reproductive status of participants. Specifically, Black women were more likely to have a microbiota containing *L. iners* (topic III) and non-*Lactobacillus* subcommunities from the first group (topics IV-A, IV-B.a and IV-B.b). Regarding differences associated with participants' reproductive state, non-*Lactobacillus* topics from the second group (topics IV-C0 and IV-C1) were more prevalent in non-pregnant individuals than in pregnant women. They were especially more frequent during menses, a time characterized by elevated vaginal inflammation, as 40% of the measured cytokines had higher concentrations during menses. In pregnant individuals, topic IV-C1 showed a strong, but not reaching significance (*p* = 0.051), association with the risk of preterm birth. It remains to be seen whether vaginal inflammation is also elevated in pregnant individuals with a higher abundance of this sub-community. Our available data did not allow us to answer this question.

As stated above, mixed membership models provide better insights into the longitudinal changes in microbiota composition than cluster membership approaches do. Another example comes from the analysis of samples from consecutive menstrual cycles. When investigating whether menstruating participants have similar microbiota variations in consecutive cycles, an analysis based on clustering membership only identified significant between-cycle correlations in two participants (10%). By contrast, the same analysis based on topic mixed memberships identified significant correlations in four participants (20%). While these results further demonstrate that topic models provide useful dimension reduction, we note that mixed membership representations may still hide important within-subcommunity variations. Here, repeating that analysis using compositional data at the taxa level showed that, in fact, 10 (50% of) menstruating participants had significant between-cycle correlations.

While the menstrual cycle appears to have a strong effect on the microbiota composition, we note that most topics or taxa reached their maximal relative abundance at different menstrual cycle phases across individuals. These inter-individual differences may be an artefact of the compositional nature of our data but could also be due to differences in ovulation timing or in hormone levels or to interactions between specific species or sub-communities. Additional studies would be necessary to disentangle these potential causes or to understand if abrupt hormonal changes, the presence of blood, or the use of menstrual protections such as pads or tampons drive the substantial changes in microbiota composition observed during menses.

Finally, to understand whether these menstrual variations in microbiota composition were accompanied by changes in the vaginal ecosystem, we analysed the vaginal pH, cytokine concentrations, and metabolite concentrations obtained from a subset of the sequenced samples. We found that the abrupt changes in microbiota composition around menses were indeed accompanied by changes in these variables. pH increased during menses in both *Lactobacillus* and non-*Lactobacillus*-dominated microbiotas, and as mentioned above, 8 out of 20 measured cytokines had elevated levels during menses (and 2 around ovulation) while 70% of the 60 metabolites that varied with the menstrual cycle peaked or dropped during menses. For example, kynurenine peaked during menses while isoleucine dropped. Kynurenine is a tryptophan catabolite via a pathway involving IDO1-mediated degradation. It is known to play a role in blood vessel dilatation during inflammatory events [26]. The elevated levels of kynurenine during menses found in our study are thus consistent with these roles and with past studies showing varying levels of kynurenine in serum and urine through the cycle [27,28]. In our vaginal samples, isoleucine, a branched-chain amino acid with important metabolic functions [29], was found with the highest levels in the luteal phase and lowest during menses. Interestingly, serum levels of isoleucine show opposite trends [30]. The menstrual changes in cytokine concentrations were consistent with those identified previously in non-pregnant individuals [31,32].

## 4. Conclusion

Topic analysis revealed bacterial sub-communities (topics) shared across pregnant and non-pregnant women, confirming the existence of sub-structures in non-*Lactobacillus*-dominated microbiota and their possible clinical relevance. Compared to clustering approaches traditionally used to categorize microbial composition, topics provide an expanded characterization of the heterogeneity of the previously described risk-associated CST IV, a high-resolution view of transitions between communities, and they better predict the loss of *Lactobacillus* dominance. We found that the menstrual cycle had a strong impact on the vaginal microbiota and on vaginal levels of 60 metabolites and half (10/20) of the measured cytokines. Of particular interest, one sub-community with increased prevalence during menses, a time of elevated vaginal inflammation, was also found to have a strong, although not quite significant (*p* = 0.051), association with the preterm birth risk. This may inspire the design of better-powered or *in vitro* studies to further investigate the functions of these sub-communities, their ecological network and their effects on the vaginal epithelium.

## 5. Material and methods

### (a) Cohorts and sample collection

#### (i) Daily samples from non-pregnant participants

Samples were obtained from 30 participants recruited at the University of Alabama, Birmingham (UAB) as part of the UMB-HMP study, which enrolled participants regardless of BV diagnosis between 2009 and 2010 [15] and in which participants with symptomatic BV were treated using standard-of-care practices [15]. The 30 participants selected for this analysis included women with stable *Lactobacillus*-dominated microbiotas, stable non-*Lactobacillus*-dominated microbiotas and unstable microbiotas. Participants self-collected daily vaginal swabs for 10 weeks, resulting in a maximum of 10 × 7 = 70 samples per individual. For further details, see [15].

#### (ii) Weekly samples from pregnant women

We used the samples from two cohorts presented previously [4]. In total, 39 pregnant individuals were recruited at Stanford University (SU), and 96 pregnant individuals were recruited at the University of Alabama, Birmingham (UAB) between 2013 and 2015. Participants from both cohorts were enrolled from the fourth month of their pregnancy (range: week 8–22), and vaginal swabs were collected weekly (approximately) until delivery with an average of 16 samples per participant and 2179 samples in total. Age, BMI and race were significantly different between the two cohorts (electronic supplementary material, table S1). Participants recruited at UAB were part of a pool of individuals for which intramuscular progesterone injections (17-OHPC) were indicated or recommended. They received that treatment throughout pregnancy with the intention of reducing their preterm birth risk. 9/39 (23%, SU) and 41/96 (43%, UAB) participants delivered preterm, defined as a delivery before 37 weeks of gestation.

#### (iii) Metabolite and cytokine samples

Metabolites and cytokine concentrations were quantified in a subset of the non-pregnant samples. Specifically, five samples separated by approximately two weeks were selected per participant. In addition, five samples each were from 10 additional non-pregnant participants of the UMB-HMP study but recruited at different sites (Emory University and the University of Maryland Baltimore). In total, metabolites and cytokines were quantified in 200 samples from 40 non-pregnant individuals.

### (b) Vaginal microbiota sequencing

#### (i) Daily samples from the 30 non-pregnant participants recruited at UAB (1534 samples)

The V3-V4 regions of the 16S rRNA gene were amplified and then sequenced with the Illumina HiSeq/MiSeq platforms.

#### (ii) Weekly samples from pregnant participants of both cohorts (SU and UAB) (2179 samples)

Raw sequence data from samples from pregnant participants were generated and processed as described in [4]. In brief, genomic DNA was extracted from vaginal samples using a PowerSoil DNA isolation kit (MO BIO Laboratories). Barcoded primers 515F/806R [33] were used to amplify the V4 variable region of the 16S rRNA gene from each sample. Pooled amplicons were sequenced on the Illumina HiSeq platforms at the Roy J. Carver Biotechnology Center, University of Illinois, Urbana-Champaign.

Demultiplexed raw sequence data from Illumina HiSeq/MiSeq were resolved to ASVs as described in the DADA2 Workflow (https://benjjneb.github.io/dada2/bigdata.html) [34].

#### (iii) Taxonomic assignment

Automated taxonomic calls were made using DADA2's implementation of the RDP naive Bayesian classifier [35] and a Silva reference database (version 132) [36]. The assignment of sequences of the most abundant ASVs were refined and standardized by using BLAST and NCBI RefSeq type strains. This is the case for *Lactobacillus, Candidatus* Lachnocurva vaginae (previously referred to as BVAB1), *Gardnerella*, and *Megasphaera lornae* species-level assignments, following recently published work on these species [37,38]. *Gardnerella* ASVs were tagged as G1, G2 or G3 *sensu* [4] based on exact matching of the ASV sequences. Taxonomic assignment tables are available (see data availability section). For downstream analyses, ASV counts were aggregated based on their taxonomic assignment.

### (c) Metabolite concentration quantification

Untargeted metabolomics was performed on 200 non-pregnant participant samples by ultra-high-performance liquid chromatography/tandem mass spectrometry (Metabolon, Inc.). Metabolite identification was performed at Metabolon based on an internally validated compound library, and results were expressed in relative concentrations, following the same protocol as in [39]. Samples were shipped and analysed in a single batch. Raw data included 853 metabolites, with, however a large proportion of missing values. Missing values may originate (i) from peak misalignment, (ii) because of concentrations lower than the detection limit or (iii) because the overall quality of a sample was low. Metabolites with missing values in more than 50% of samples were excluded from the analysis (removing 517 metabolites). Samples with more than 60% missing data for the remaining 336 metabolites were further excluded. Raw metabolite relative concentrations were transformed using a variance stabilizing method [40].

### (d) Cytokine concentration quantification

Vaginal cytokines were quantified in the 200 non-pregnant participant samples using a Luminex-based assay with a custom kit of 20 analytes (IFN*γ*, IL-1α, IL-1β, IL-4, IL-5, IL-6, IL-8, IL-10, IL-12p70, IL-13, IL-17, IL-21, IL-23, IP-10, ITAC, MIG, MIP-1α, MIP-1β, MIP-3α and TNF*α*) following the same protocol as in [12]. The assay was run on a Luminex FLEXMAP three-dimensional instrument. Measurements below the limit of quantification for a given cytokine were imputed at half the lower limit of quantification (LLOQ/2). Measurements above the limit of quantification for a given cytokine were imputed as equal to the upper limit of quantification (ULOQ). Values reported here represent medians of two technical replicates, calculated after imputation. Missing cytokine values (11/4000 = 0.275%) represent technical failures of the assay for that analyte. Concentrations were log-transformed for downstream analyses.

### (e) Integration into a multi-assay experiment object

All analyses were performed in the R software environment [41]. Packages used for the analyses are referred to in the next sections. Raw datasets were loaded and minimally processed before being formatted into SummarizeExperiment objects [42], then combined into a single S4 object using the MultiAssayExperiment package [43].

### (f) Identifying bacterial sub-communities using topic analysis

Microbial communities were estimated using LDA models [21,22]. Models were fitted to the data for K (the number of topics) = 1 to 25 using the R package ‘topicmodels’ [44]. Models were fitted on the taxonomically agglomerated ASV counts directly, without any prior normalization; the library size being one of the parameters of this Bayesian framework. Topics were aligned across K using the alto package and topic alignment method described in [23]. Optimal K was chosen to maximize topic coherence score [23].

### (g) Comparison of topic and sub-CST composition and sample assignment to sub-CST

Both sub-CSTs centroids [19] and topics are compositional (proportions sum to 1 per sub-CST/topic). They were compared based on their pairwise Bray–Curtis dissimilarity. Prior to computing their similarity, we harmonized taxonomic assignments using the ValenciaR package. For example, sub-CST taxonomy does not differentiate between *Gardnerella* species so *Gardnerella* topic proportions were aggregated. Samples were assigned to the sub-CST that maximizes the Yue and Clayton similarity between the sample composition and the sub-CST centroids, as per [19].

### (h) Microbiota composition prediction from sub-CST and topic membership

To evaluate how well sample composition was represented by sub-CST categories (fixed composition) or topics (fewer topics than sub-CSTs, but mixed memberships), we compared the Bray­–Curtis dissimilarity between the actual sample compositions and those predicted by topic or sub-CST membership(s). For sub-CST, the sample's predicted composition is the composition of its sub-CST centroid. For topics, it is the average of topics composition (displayed in figure 2*b*) weighted by the proportion of each topic in that sample (i.e. ${\hat{\;p}}_{i,j}\;=\sum_{k\;=\;1}^{K}\gamma_{i,k}\;\;\beta_{k,j}$ where ${\hat{\;p}}_{i,j}$ is the predicted proportion of taxa *j* in sample *i*, *k* the topic index, $\gamma_{i,k}$ the proportion of topic *k* in sample *i* and $\beta_{k,j}$ the proportion of taxa *j* in topic *k*).

### (i) Microbiota local stability

Samples were classified as belonging to a stable microbiota if they were part of a series of five consecutive samples with a Bray–Curtis dissimilarity smaller than 0.25 (0.15 and 0.35 also considered in sensitivity analysis). Otherwise, the microbiota was considered unstable.

### (j) Predicting the risk of losing lactobacillus dominance

To predict the risk of losing *Lactobacillus* dominance at the next time-point in participants' longitudinal time series, logistic regression models were fitted to the data. Explanatory variables were the sample sub-CST or the sample topic proportion at the current time point. The response variable was a binary variable indicating if the next sample had greater than 50% *Lactobacillus* (dominance). Models were fitted on a training set (a random sample comprising 80% of the total dataset) and prediction performances evaluated on the remaining 20% of the dataset. The procedure was repeated independently 10 times. Because the loss of *Lactobacillus* dominance is rare (approx. 10% of cases), we weighted the sample to give more weight (10-fold) to the minority class when training the models, and used the F1 score (harmonic mean between precision and sensitivity) for performance evaluation. Differences in the sub-CST- versus topic-based prediction performances were tested with a Wilcoxon rank sum test.

### (k) Associations between topic composition and demographic variables

A Dirichlet regression was used to test if race, study site, or pregnancy were associated with differential topic proportions. Because most participants’ race was Black or White, we defined a three-category variable: Black, Other and White (‘Other’ served as reference). Pregnancy and site were binary variables (pregnant versus non-pregnant and SU versus UAB). The model is $\;p=\beta \;+\;\alpha_{R}R\;+\;\alpha_{P}P\;+\;\alpha_{S}S\;+\varepsilon \;$where ***p*** is the vector of topic proportions lying on the K-dimension simplex. Coefficients were obtained using the DirichletReg package in R [45].

### (l) Identification of phases of the menstrual cycle

Menstrual cycles were identified from bleeding flows reported daily by participants on a scale from 0 (none) to 3 (heavy). A hidden semi-Markov model was specified to account for empirically observed distributions of cycle length and bleeding patterns across the menstrual cycle, including spotting between menses [46]. Data of participants who reported too few days with bleeding (i.e. less than 3/70 study days) or too many (i.e. more than 30/70 study days) were excluded from the menstrual cycle analyses. To allow for between cycle comparisons and account for variable cycle lengths, menstrual timing was standardized following recommendations for studying menstrual cycle effects [25]. These recommendations account for well-documented larger variations in follicular phase durations than in luteal phase durations and optimally align ovulation across cycles in the absence of hormonal and/or ovulation markers. In brief, once cycles were identified (see electronic supplementary material, figure S9), days were numbered forwards and backwards from the first day of the period. Cycles were then standardized from day −18 (i.e. 18 days before menses) to day +7 (i.e. 7 days after the first day of menses).

### (m) Testing for differential abundance throughout the menstrual cycle

To identify metabolites, cytokines or topics with differential abundance (metabolites or cytokines) or differential probabilities of being present at specific phases of the menstrual cycle, a linear model (for abundances) or logistic regression (proportions) was fitted to circular splines parameterized with 4 d.f. (R package ‘pbs’). ANOVA *p*-values were corrected for multiple testing using the Benjamini–Hochberg method.

### (n) Associations between topic proportions and preterm birth

To test if topic proportions were associated with preterm birth, a logistic regression model was fitted on the data. Explanatory variables were the per-participant topic proportion averages, and the response variable was a binary variable indicating whether participants delivered preterm or not.

### (o) Correlation in vaginal microbiota composition between two consecutive cycles

To evaluate how the menstrual cycle affects the vaginal microbiota composition, we computed the RV coefficient [47] and associated permutation test *p*-value [48] between the topic or taxa proportions of the first cycle and of the second cycle. To quantify the magnitude of change in microbiota composition throughout the cycle (*x*-axes of figure 4*b*), we first computed the average topic or taxa proportion across cycles for each cycleday. Then, pairwise Bray–Curtis dissimilarities were computed so that the average compositions of each cycleday were compared against each other. The maximum value was used to quantify the magnitude of change throughout the menstrual cycle for each participant.

## Preprint servers

This manuscript was deposited on bioRxiv (https://www.biorxiv.org/content/10.1101/2021.12.10.471327v1) under a CC-BY-ND 4.0 International licence.

## Data Availability

Sequence data for samples from non-pregnant study participants are available in the NCBI Sequence Read Archive (SRA) under BioProject accession numbers PRJNA208535 (samples beginning with UAB) and PRJNA575586 (samples beginning with AYAC and EM). Sequence data from samples from pregnant study participants are available on the SRA (accession no. PRJNA393472). Raw data and R code enabling the reproduction of the analyses are available at https://purl.stanford.edu/gp215vr4425. Supplementary material is available online [49].
